# Early in-session predictors of response to trauma-focused cognitive therapy for posttraumatic stress disorder

**DOI:** 10.1016/j.brat.2015.10.001

**Published:** 2015-12

**Authors:** Francesca Brady, Emma Warnock-Parkes, Chris Barker, Anke Ehlers

**Affiliations:** a Research Department of Clinical, Educational and Health Psychology, University College London, Gower Street, London, WC1E 6BT, UK; b University of Oxford, Department of Experimental Psychology, South Parks Road, Oxford, OX1 3UD, UK; c NIHR Oxford Cognitive Health Clinical Research Facility, UK; d King's College London, Department of Psychology, Institute of Psychiatry, De Crespigny Park, London, SE5 8AF, UK; e NIHR Biomedical Research Centre for Mental Health, South London and Maudsley NHS Trust and King's College London, UK

**Keywords:** Posttraumatic stress disorder, Cognitive therapy, Treatment outcome, Therapeutic alliance, Perseveration, Introspection

## Abstract

Trauma-focused cognitive behaviour therapy is effective in treating posttraumatic stress disorder but non-response rates range between 25% and 50%. Results of previous research on patient characteristics predicting outcome are inconsistent and mainly focused on demographic and diagnostic variables. This study examined whether behavioural predictors of poor treatment response can be observed in early sessions. It was predicted that greater patient perseveration, lower expression of thoughts and feelings and weaker therapeutic alliance would be associated with poorer outcomes. We also explored the relationships of patient behaviours with therapeutic alliance and the efficiency and competence of treatment delivery. Audio or video recordings of the initial treatment sessions of 58 patients who had shown either good (n = 34) or poor response (n = 24) to cognitive therapy for PTSD (CT-PTSD, Ehlers & Clark, 2000) were blindly coded for patient perseveration, expression of thoughts and feelings, therapeutic alliance, efficiency and competency of treatment delivery and therapist competence. Poor responders showed more perseveration and less expression of thoughts and feelings in the initial session. Patient perseveration and low expression of thoughts and feelings were associated with poorer therapeutic alliance and compromised treatment delivery. Patients with these behavioural characteristics may benefit from additional treatment strategies. Limitations of the study and implications for clinical practice are discussed.

## Highlights

• The study examined whether patient behaviour in initial treatment sessions predicts outcome.
• Poor treatment responders showed more perseveration and lower expression of thoughts and feelings than good responders.
• These patient characteristics were associated with poorer therapeutic alliance.
• These characteristics were also associated with lower ratings of the efficacy and competency of treatment delivery.

## 1 Introduction

Trauma-focused cognitive behaviour therapy (CBT) is an effective first-line treatment for posttraumatic stress disorder (PTSD) (Bradley et al., 2005, Cloitre, 2009, National Institute for Health and Care Excellence, 2005). However, treatment non-response rates range between 25% and 50% (for reviews see Bisson et al., 2013, Bradley et al., 2005, Schottenbauer et al., 2008). There is a need to examine patient factors that might be predictive of poorer therapeutic response (Schottenbauer et al., 2008), as this may help refine treatment procedures or identify patients in need of additional interventions. Evidence regarding patient factors associated with poor treatment outcomes in CBT for PTSD is limited and there is a need for further research to examine indicators of non-response to treatment (Hembree, Marshall, Fitzgibbon, & Foa, 2001).

### 1.1 Patient factors predicting treatment response

It has been argued that patient factors account for between 40% and 87% of the variance in treatment outcome (Bohart and Greaves Wade, 2013, Lambert, 1992, Wampold, 2010). For PTSD treatment, demographic and diagnostic variables, including gender, age, ethnicity, comorbid psychiatric diagnoses and trauma characteristics, predicted outcome in some studies. However, few variables consistently predicted outcome across studies (e.g., Ehlers et al., 2013, Schottenbauer et al., 2008, van Minnen et al., 2002).

There has also been some exploration of patient cognitions and behaviours that may moderate response to treatment. This line of research may be helpful in identifying variables that are malleable with targeted intervention. Recurrent and repetitive negative thinking about the trauma and its consequences has been hypothesised to maintain PTSD symptoms (Ehlers and Clark, 2000, Ehlers and Steil, 1995). This can take the form of rumination about what happened and worry about potential future trauma. Rumination is distinct from intrusive re-experiencing as ruminative thoughts are longer in duration and involve evaluative verbal thoughts rather than sensory responses and memories (Ehring et al., 2008b, Speckens et al., 2007). Rumination and worry often overlap and co-occur (Fresco, Frankel, Mennin, Turk, & Heimberg, 2002) and consequently researchers have used the terms “repetitive thought” or “perseverative thinking” to capture the process of perseverative thinking about the past or future (Segerstrom et al., 2003, Watkins, 2008). There is evidence that “perseverative thinking” is a transdiagnostic process that is involved in several disorders including PTSD (Ehring & Watkins, 2008).

Several prospective studies found that perseverative thinking about the trauma and its consequences in the initial weeks after trauma predicted chronic PTSD (Ehring et al., 2008a, Kleim et al., 2007, Michael et al., 2007). Perseverative thinking may maintain PTSD symptoms in several ways, including: inducing a persistent state of negative emotional arousal (Ehring et al., 2009, Moore et al., 2008), strengthening problematic appraisals such as excessive self-blame and perceived permanent change, preventing elaboration of trauma memories and providing retrieval cues for intrusive memories (Ehlers & Clark, 2000).

It has been suggested that the presence of perseverative thinking may prevent the patient from receiving a sufficient “dose” of therapy (Echiverri et al., 2011, Wells and Sembi, 2004). Echiverri et al. (2011) presented the case of a non-responder to prolonged exposure. They reported that the patient's in-session rumination facilitated avoidance of engaging with emotions present during their trauma (i.e. fear). The authors proposed that this prevented habituation during repeated exposure and thus blocked integration of corrective information into the trauma memory, leading to a poor treatment outcome. However, as this was a single case study, the authors highlighted the need for further therapy process research, particularly comparison of treatment responders with non-responders. The present study investigated the influence of observed perseveration on the efficiency and competency of treatment delivery, therapeutic alliance and treatment outcome in another form of trauma-focused CBT, CT-PTSD (Ehlers and Clark, 2000, Ehlers et al., 2005).

There is a long tradition in patient-centred and psychoanalytic psychotherapy research of investigating the role of patients' degree of self-exploration, introspective awareness or psychological mindedness (i.e. the ability to discriminate and describe emotions and thoughts), on the therapeutic relationship, commitment to psychotherapy and treatment outcome. There is evidence of a positive association between psychological mindedness and treatment outcome (Conte et al., 1990, Piper et al., 1994). Alexithymia, a personality trait characterised by poor insight into and expression of personal emotional experience, has been associated with PTSD (see Frewen, Dozois, Neufeld, & Lanius, 2008 for a review), and also linked to poor treatment outcome in psychotherapy (McCallum et al., 2003, Ogrodniczuk et al., 2005).

These variables have received less attention in CBT. Two studies investigating alexithymia and CBT treatment for sub-threshold depression and obsessive compulsive disorder did not find a relationship with outcome (Rufer et al., 2004, Spek et al., 2008). There is some evidence that psychological mindedness improves during CBT (Nyklíček Majoor & Schalken, 2010). In cognitive behavioural treatments of PTSD, lower expression of thoughts and feelings early in therapy may interfere with establishing a good therapeutic relationship or make it harder for the therapist to identify cognitions that maintain a patient's problem and tailor interventions accordingly. The ability to differentiate emotions has predominantly been assessed using self-report measures and it has been argued that behavioural observation may be a more reliable assessment (Kashdan, Barrett, & McKnight, 2015). In line with this, the present study explored behavioural indicators of introspective awareness (expression of thoughts and feelings) in an early PTSD treatment session and their relationship with treatment outcome, efficiency and competency of treatment delivery and the therapeutic relationship.

### 1.2 Therapeutic alliance

There is a large body of literature relating to the effects of therapeutic alliance on treatment outcome (Baldwin & Imel, 2013), but little research has focused on these factors in PTSD specifically. A good therapeutic alliance has been demonstrated to be important to the outcomes of cognitive behavioural therapy in general (Hardy, Cahill, & Barkham, 2007) and some evidence indicates that strong early alliance can predict good treatment compliance and outcome (Keller et al., 2010, Klein et al., 2003). However, the overall relationship between alliance and treatment outcome may only be small (Crits-Christoph, Connolly Gibbons & Mukherjee, 2013). Moreover, in a study of patients with depression DeRubeis and Feeley (1990) found that a strong therapeutic alliance in fact followed symptomatic change, rather than preceding it (see also DeRubeis, Gelfand, German, Fournier, & Forand, 2014, for a detailed discussion of temporal confounds in process-outcome research). Webb et al. (2011) concluded that the “bond” component of the alliance might be dependent on prior symptomatic improvement, suggesting the relationship between alliance and treatment outcomes may not be unidirectional. McLaughlin, Keller, Feeny, Youngstrom, and Zoellner (2014) found that unrepaired ruptures in alliance were predictive of poor outcome in prolonged exposure for PTSD. Thus, alliance may change over the course of therapy and the pattern of changes may be relevant for treatment outcome.

### 1.3 Treatment delivery

The competence and efficiency with which treatment is delivered may predict outcomes. For PTSD, Ehlers et al. (2013) and Duffy, Gillespie, and Clark (2007) found a higher drop-out rate for inexperienced therapists. Overall the size of the relationship between competency and treatment outcomes varies across studies (Branson et al., 2015, Strunk et al., 2010, Webb et al., 2010), and depends on the range of competency considered. The present study explored whether patient behaviours may affect the competency and efficiency of treatment delivery in experienced therapists.

### 1.4 Observations of in-session behaviours

Most previous research on the influence of patient characteristics on treatment outcome studied either self-reports or demographic and diagnostic predictors. Systematic observation of therapy sessions might be fruitful in establishing behavioural characteristics of patients and their interaction with the therapist that are predictive and could guide the therapist in tailoring the treatment to the patient's behaviours. Few studies to date have undertaken this type of analysis. For example, Darcy et al., (2013) viewed videotapes of family therapy sessions for adolescents with anorexia and established potential in-session behaviours which were related to treatment outcome. With regards to PTSD, Ready et al. (2015) utilised a similar observational methodology (using an adapted pre-existing coding system) to examine changes in the cognitions of children and adolescents undergoing trauma-focused CBT.

### 1.5 Aims of current study

The aim of this study was to identify observable patient behaviours in early treatment sessions that predict poor response to CT-PTSD. The study compared blind ratings of recordings of the first or, if this was not available, the second therapy session of good and poor treatment responders.

Following previous research, it was predicted that poor treatment responders would show a higher degree of perseveration and less expression of thoughts and feelings in the initial sessions than good responders. We also expected that the therapeutic alliance would be weaker for poor responders. We further investigated the relationship of these factors with the efficiency and competency of treatment delivery.

## 2 Method

This study used a two group design, comparing good and poor responders to treatment.

### 2.1 Participants

The study used video or audio recordings of therapy sessions of patients who had received a course of trauma-focused cognitive therapy for PTSD (CT-PTSD, Ehlers and Clark, 2000, Ehlers et al., 2005) at a National Health Service clinic in South London, UK, for index traumas that occurred in adulthood. Patients met DSM-IV diagnostic criteria for PTSD according to the Structured Clinical Interview for DSM-IV (First, Spitzer, Gibbon, & Williams, 1996). For the purposes of this study, we were interested in patients who had attended at least 5 treatment sessions (to make sure that they had at least some experience with the treatment procedures beyond clinical assessment), with a therapist who was a qualified clinical psychologist or nurse therapist and had completed training in CT-PTSD (to make sure treatment was delivered competently), and either showed a poor response (defined as a reduction in PTSD symptoms of no more than a third) or good response (defined as a reduction defined as a reduction in PTSD symptoms of two thirds or greater). Outcome measures at initial assessment and the last treatment session were available for all patients.

First, tapes were drawn from a consecutive sample of 330 patients. Detailed outcomes are described in Ehlers et al. (2013). Of these, 62 (19%) were training cases and a further 29 (8.8%) dropped out before receiving 5 sessions. Of the remaining eligible 245 patients, 68 (27.8%) met criteria for poor response and 117 (47.8%) criteria for good response.^1^ We then checked whether a working video or audio recording of the first or second session was available for these patients. As this selection procedure did not yield enough working tapes,^2^ a further 83 recent cases were screened against the same criteria to identify further working tapes.

Overall, 25 working video or audio recordings of the first therapy sessions for poor responders were identified. One of these sessions focused exclusively on ‘imaginal reliving’ of the trauma and was excluded, as it restricted the opportunity to observe the variables of interest, resulting in 24 tapes that were included in the study. A total of 68 tapes of good responders were identified, and 34 tapes were randomly selected for the study. Thus, a total of 58 tapes were included in the study.

In the sample as a whole, the mean age was 39.2 years (SD = 11.4) with 25 (43%) female participants. In terms of ethnicity, 33 participants (57%) were of white origin, 16 (28%) were of black ethnic origin and nine (15%) came from another ethnic group. Regarding marital status, 25 (45%) were married or cohabiting, 26 (45%) had never married and six (10%) were divorced, separated or widowed. As for educational background, 27 participants (47%) had left education at age 16 (after attaining GCSEs), eight (14%) after completing A-levels at age 18 and 18 (31%) after university with five (9%) reporting they had not completed any school qualifications. In terms of the type of presenting index trauma, 36 (62%) had experienced interpersonal violence, 16 (28%) had been involved in an accident, three (5%) had witnessed harm to another person and three (5%) had experienced another type of traumatic event. Participants had experienced a mean of 2.61 (SD = 2.24) traumas other than the index trauma. The observed sessions lasted between 50 and 116 min (M = 82.26, SD = 16.80). See Table 1 for demographic information for the good and poor outcome groups.

### 2.2 Treatment

CT-PTSD (Ehlers et al., 2005) is based on Ehlers and Clark's (2000) model of PTSD. The key aims of therapy are to: identify and address trauma-related negative appraisals; update trauma memories; discriminate triggers of intrusions; and change unhelpful cognitive and behavioural coping strategies that maintain a sense of current threat (for details of treatment procedures see http://oxcadat.psy.ox.ac.uk/downloads/CT-PTSD%20Treatment%20Procedures.pdf/view). The initial treatment session aims to normalise PTSD symptoms, agree on treatment goals and start to develop an individualised case formulation on the basis of Ehlers and Clark's (2000) model and rationale for treatment. This includes a first exploration of what happened during the trauma, personal meanings of the trauma and its consequences, and the content of the patient's intrusive memories and their triggers. A thought suppression experiment introduces the idea that the way the patient has tried to cope with the intrusions has unwanted effects, and a rationale for working on the trauma memories and their triggers is given. In addition, the therapist encourages re-engagement with daily or social activities that may have become restricted since the traumatic event and agrees first “reclaiming your life” assignments with the patient.

Therapists who provided tapes for the study were qualified clinical psychologists (n = 12), or nurse therapists (n = 2) who had completed training in CT-PTSD.

### 2.3 Measures

#### 2.3.1 Treatment outcome

Outcome was assessed with the *Posttraumatic Stress Diagnostic Scale* (PDS; Foa, Cashman, Jaycox, & Perry, 1997). This scale asks patients to rate the frequency of 17 PTSD symptoms specified in DSM-IV from 0 (“never”) to 3 (“3–5 times a week/almost always”). The sum score is a measure of PTSD severity. The PDS has been demonstrated to be reliable and valid (Foa et al., 1997) in measuring current PTSD symptoms.

#### 2.3.2 Patient predictors

A coding frame and manual for patient behaviours were developed for the purposes of this study.^3^ The literature review indicated that client perseverative thinking, expression of thoughts and feelings and therapeutic alliance would be candidate predictors of outcome. In line with the procedures used in similar studies (Darcy et al., 2013); Ready et al., 2015), this was followed by a discussion with three specialist therapists about their clinical experience, and viewings of recordings of known responders and non-responders to treatment from previous research trials, and the development of a coding scheme. Likert rating scales were chosen after initial exploration of alternative scorings methods such as frequency ratings as this led to the best agreement between raters. Descriptors were developed for each level of the scale for each item. The coding frame was piloted using session recordings from previous trials and the manual and coding frame descriptors were then adapted according to feedback from this process. Following the development of the coding frame, a clinical psychologist (FB) rated the 58 tapes without knowledge of treatment outcome. An expert clinician (EWP) independently rated 10 (17%) of the session recordings, without knowledge of treatment outcome. Inter-rater reliability was calculated with intraclass correlations (see Landis & Koch, 1977).

##### 2.3.2.1 Patient perseveration

Indicators of patient perseverative thinking were: The patient (1) is preoccupied with particular themes and returns to them repeatedly, (2) asks “what if/why” questions that are unproductive and do not lead to additional insight, (3) does not respond to therapist's attempt to move on to another topic, or (4) provides excessive detail or multiple examples in response to the therapist's questions. Examples are given in the coding manual, such as “why did this happen to me?” and “what if this therapy doesn't work?”. The extent to which the patient demonstrated these behaviours was rated on a seven-point Likert scale, from 0 indicating perseveration was not observed to 6 indicating perseveration was always present during the session. There was very good inter-rater agreement, as indicated by an intraclass correlation of .87.

##### 2.3.2.2 Patient expression of thoughts and feelings

Indicators of *introspective awareness* were utterances indicating that the patient was aware of their internal experiences and could describe them, i.e. could identify their thoughts, emotions or physical sensations and make links between them. Examples are given in the coding manual, such as “I thought I was going to die and I felt really frightened”, “I keep dwelling on what happened and it makes me feel really down”. The degree to which patients showed these behaviours was rated on a seven-point Likert scale, from 0 indicating no expressions of internal experiences were observed to 6 indicating that internal experiences were always expressed during the session. There was very good inter-rater agreement, as indicated by an intraclass correlation of 0.88.

#### 2.3.3 Therapeutic relationship

##### 2.3.3.1 Working alliance

This was assessed with the Inventory-Observer Version-Short Form (WAI-O-S; Horvath and Greenberg, 1986, Tracey and Kokotovic, 1989). This scale is a 12-item measure of the quality of therapeutic alliance, adapted to be rated from an observer perspective. Each item is rated on a seven-point Likert scale with graded descriptors, following a format utilised by Berk, Safran, Muran, and Eubanks-Carter (2010). There are two reverse scored items. Following recommendations for the analysis for CBT sessions by Andrusyna, Tang, DeRubeis, and Luborsky (2001), we calculated separate scores for the factors Agreement/Confidence (9 items) and Relationship (3 items). Scores are totalled with higher scores indicating a greater observed alliance. Good reliability has been demonstrated for the WAI-O-S (r = 0.81; Gelfand & DeRubeis, undated, cited in Andrusyna et al., 2001) and research demonstrates support for the validity of the Working Alliance Inventory (Horvarth, 1994). Inter-rater reliability for the WAI-O-S total score was very good, as indicated by an intraclass correlation of .91.

#### 2.3.4 Treatment delivery

##### 2.3.4.1 Competency of treatment delivery

Competency was assessed with an adaptation of the Cognitive Therapy Scale-Revised (CTSR; Blackburn, James, Milne, & Reichelt, 2001). Blackburn et al. (2001) have demonstrated good reliability and validity for the CTSR. For PTSD, a specific version of the CTSR has been used in previous trials (Ehlers et al., 2014) that consists of 9 generic CBT competency skills and 7 additional items pertaining to specific techniques utilised in CT-PTSD. Of these, only 3 apply to the first treatment session (PTSD conceptualisation (formulation), psychoeducation about symptoms and discussion of “reclaiming your life” activities) and were therefore included in the scoring. The mean total score of the 9 general and 3 specific items was used to measure competency of treatment delivery. A score of 3 is considered satisfactory, and scores of 4–6 indicate good to excellent competency. Inter-rater reliability showed very good agreement (intraclass correlation of .84). As to be expected for experienced therapists, the mean competency was good to very good and the range of competency ratings small (mean = 4.11, SD = 0.88).

##### 2.3.4.2 Efficiency of treatment delivery

The treatment manual describes 14 treatment elements that are covered at the start of therapy in the first one to two sessions (as previously described). The rater recorded how many of these were covered in the session, and the percentage of completed elements was calculated as an index of the efficiency of treatment delivery. Inter-rater reliability showed very good agreement (intraclass correlation of .93). On average, the sessions covered 61.59% of the elements, SD = 18.74.

### 2.4 Procedure

The study was approved by the local ethics committee. Patients had received an information sheet before treatment and had given written consent. The raters were blind to the treatment outcome status of the patient.

### 2.5 Data analysis

Data were analysed using SPSS statistical software, version 22. Group comparisons on demographic and diagnostic variables used independent t-tests for continuous and Chi–Square tests for categorical variables. The main group comparisons on the dependent variables where calculated with ANCOVAs, controlling for initial PDS, comorbid anxiety and depression, psychotropic medication use, length of treatment session and marital status. Kolmogorov–Smirnov tests showed that all but one dependent variable (treatment efficiency in the good responders group) was normally distributed. Inspection of the frequency histogram indicated the data for this variable were not skewed or bimodal and only deviated slightly from normality. Thus, parametric tests were used to analyse the data. As there was no existing study of behavioural indices of perseverative thinking on therapy outcome other than Echiverri et al. (2011), we based our power analysis of the required sample size on the very large effect of rumination as a predictor of chronic PTSD (Ehring et al., 2008a, Ehring et al., 2008b, Kleim et al., 2007). Power analyses using G-Power (Faul, Erdfelder, Buchner, & Lang, 2009) showed (with α = .05, d = .8 and 80% power) indicated that 52 participants in total were needed to detect such an effect.

## 3 Results

### 3.1 Demographic and diagnostic variables

Table 1 shows demographic and diagnostic variables for the good and poor outcome groups. Patients with poor outcome had higher baseline PDS scores and were more likely to have a comorbid axis 1 disorders and to be taking psychotropic medication. They were less likely to be married or be living with a partner and had marginally shorter treatment sessions. ANCOVAs were used to control for these variables when testing for group differences on the dependent variables.

### 3.2 Group differences between good and poor responders

Table 2 shows the scores for good and poor responders for patient perseveration and expression of thoughts and feelings and for therapeutic alliance. ANCOVAs showed that poor responders displayed more in-session perseveration and less expression of thoughts and feelings than those with a good treatment response. The effect size of the group differences in both patient behaviours was large. There was also a group difference in the agreement factor of therapeutic alliance, with a medium to large effect size, but no group difference in the relationship factor. Mean scores indicated that alliance was strong across both groups.

### 3.3 Correlational analyses

Table 3 shows the results of the correlational analyses. Patient perseveration was not associated with expression of thoughts and feelings (r = −.19, p = .16). Both patient factors correlated with the agreement factor of working alliance. There was a moderate negative correlation with perseveration and a large positive correlation with expression of thoughts and feelings. Greater expression of thoughts and feelings also related with better alliance in the relationship factor. Both patient factors also correlated with the quality of treatment delivery. Perseveration showed large negative correlations with ratings of treatment efficiency and competency of delivery. Expression of thoughts and feelings showed a moderate correlation with competency of delivery.

## 4 Discussion

The results showed that, as hypothesised, patients who obtained little benefit from CT-PTSD showed greater perseveration in their initial therapy sessions than those with good treatment outcome. The findings are in line with the case study of prolonged exposure by Echiverri et al. (2011). If replicated, these findings suggest that perseverative thinking may interfere with treatment response to trauma-focused cognitive behaviour therapy.

There are several mechanisms by which perseveration could interfere with progress in CT-PTSD. First, valuable time may be taken up in the session so that there is less time for core treatment procedures (Echiverri et al., 2011, Wells and Sembi, 2004). There was some evidence for this suggestion, as a negative correlation indicated that the percentage of the planned session content covered correlated with lower patient perseverative thinking. Thus, patients may get a lower dose of the effective ingredients in therapy in the allocated time. Second, maladaptive perseverative thinking tends to be abstract (Watkins & Moulds, 2005) and abstract thinking about one's problems may make it harder for the patient and therapist to agree on concrete steps to take in therapy and build a constructive collaborative relationship. The result that patient perseveration in the session was associated with a weaker agreement/confidence aspect of therapeutic alliance is in line with this hypothesis. Third, perseveration may also affect therapist behaviour and perceptions, and repeated failed attempts to steer the patient away from repetitive thinking towards the goals for the session may negatively impact on therapists' confidence and performance. In line with this hypothesis, perseveration was also associated with lower ratings of the competency of treatment delivery. However, these results are based on patterns of correlations and causal inferences cannot be drawn.

In line with findings for other psychotherapies (Conte et al., 1990, McCallum et al., 2003, Ogrodniczuk et al., 2005, Piper et al., 1994) patients with poorer treatment outcome were less able to articulate their thoughts, feelings and body sensations and make links between these in the initial session than those with good outcome. These findings were independent of individual differences in perseveration. Several mechanisms are conceivable. First, low expression of thoughts and feelings may make it harder for the therapist to tailor interventions to the individual's pattern of emotions and cognitions (e.g., address guilt, anger or shame effectively). The moderate correlation with competency of delivery is in line with this suggestion. Future studies could explore this hypothesis by coding the content of cognitions and emotions expressed. Second, low expression of thoughts and feelings in the session may indicate high levels of cognitive avoidance that may interfere with accessing moments in the memory of trauma that carry problematic meanings (Ehlers & Clark, 2000). Third, it may affect the quality of therapeutic relationship. The high correlation with both factors of therapeutic alliance is in line with this hypothesis, although the correlational nature of the data precluded causal inferences.

The results supported for the role of the therapeutic alliance in treatment outcome observed for other treatments (Baldwin & Imel, 2013). However, the results were dependent on which factor of alliance was considered. In line with Andrusyna et al. (2001), there was a significant group difference in the alliance agreement/confidence factor, but not in the relationship factor. Thus, while the overall emotional relationship between patients and therapists were rated as high regardless of subsequent treatment response, a better quality of the collaborative working relationship observed in the first sessions was related to better outcome. Overall, these results are in line with previous work showing that therapeutic alliance may account for around 5% of the variance in overall treatment outcomes (Crits-Christoph et al., 2013). The range of alliance ratings may have been restricted as the mean ratings of alliance were high, and studies using a wider range of therapists with different levels of experience may find stronger effects.

A strength of this study is that observable patient behaviour in the therapy session was assessed with reliable rating scales, and that treatment outcome was related to patient behaviour in the initial sessions. This supports the practical relevance of the findings.

However, there are also several limitations. First, as this study was restricted to observer-rated data, future research would benefit from using both self- and observer-rated measures in order more fully understand the internal processes that accompany the behaviours under examination. Second, the study relied on the availability of working recordings of the initial sessions. Unfortunately, such recordings were only available for less than half of the eligible patients. It is unlikely though that this introduced any systematic bias that would have influenced the pattern of results. Third, the definition of poor and good outcome was arbitrary and chosen to avoid overlap in outcome between the groups, while ensuring that sufficient tapes could be identified. It is conceivable that stricter or more lenient criteria for poor outcome would have led to somewhat different findings. Fourth, the study relied on tapes from sessions with therapists who were experienced in delivering CT-PTSD. While this facilitated the study of patient behaviours and reduced error variance due to therapist behaviour, it restricted the range of competency ratings and made it impossible to systematically study therapist effects (see DeRubeis et al., 2014, for a detailed discussion of the role of the range in therapy quality and patient characteristics on associations with outcome). It may also have reduced the variance in the therapeutic alliance measure so that the effects of the therapeutic relationship on outcome may have been underestimated. Furthermore, as it is likely that in-session factors such as alliance may vary during treatment and across patients, rating alliance over multiple sessions may show a clearer pattern of results (Crits-Christoph et al., 2013, DeRubeis et al., 2014). Fifth, this study concentrated on patients who attended at least 5 treatment sessions and thus had at least some experience with the treatment. It is possible that the client factors investigated in this paper are also predictive of failure to engage with treatment and early attrition. Future studies are needed to investigate this possibility. Finally, the ratings of patient behaviours, alliance and therapist competence were not independent as they were obtained from the same rater. We therefore cannot rule out that this may have influenced some of the correlations observed in this study. For future studies, it would be preferable to rate patient and therapist behaviour independently (Baldwin, Wampold, & Imel, 2007).

### 4.1 Clinical implications

One of the major clinical implications of this study is for the need for clinicians to identify perseverative thinking and to address these issues directly with the client early on in treatment. This would ensure that time is used productively and that clients are effectively emotionally engaged, in order that processing of adaptive information into the trauma memory can occur. Early identification and regular monitoring during therapy of perseverative thinking might also anticipate and avoid any ruptures in the therapeutic alliance. As this study indicates perseverative thinking might have implications for clients not receiving the full “dose” of treatment, improving both therapist and client awareness of perseverative thinking might also encourage closer monitor of adherence to a session plan and the treatment manual, in order to ensure effective and efficient treatment delivery. As low client awareness and/or ability to articulate of one's thoughts and feelings may interfere with building a productive therapeutic relationship, it may also be important for clinicians to identify and facilitate this process in early treatment sessions. As it is possible that several other factors influence a client's ability to express their thoughts or feelings, future studies should test whether additional interventions specifically addressing client perseveration and/or identification and expression of thoughts and feelings are helpful in improving outcomes.

## 5 Conclusions

The results of this study showed that patient perseveration and low expression of thoughts and feelings observed in early therapy sessions were associated with poorer response to trauma-focused cognitive therapy for PTSD. The present correlational data suggest that they may operate by different mechanisms. Future research should attempt to monitor these factors over the course of therapy with a view to clarifying their relationship with poor treatment response. One of the key implications of this study is to highlight the importance of clinicians' identifying perseveration early on in treatment, in order to help patients gain full benefit from trauma-focused cognitive therapy.

## Figures and Tables

**Table 1:** Sample characteristics.

Variable	Good responders (*n* = 34)		Poor responders (*n* = 24)		*t/χ*^*2*^*(df)*	*p*
*M (SD)*	*N (%)*	*M (SD)*	*N (%)*
Age (in years)	40.5 (11.9)		37.4 (11.6)		t (56) = 1.02	.312
Gender						
*Female*		16 (47.1)		9 (37.5)	*χ*^*2*^ (1^c^) = 0.52	.469
*Male*		18 (52.9)		15 (62.5)
Marital status					*χ*^*2*^ (2^c^) = 6.63	.036^b^
*Married*		20 (58.8)		6 (25.0)
*Never married*		11 (32.4)		15 (62.5)
*Previously married*		3 (8.8)		3 (12.5)
						
Ethnic Background					*χ*^*2*^ (2^c^) = 0.78	.676
*White*		20 (58.8)		13 (54.2)
*Black*		8 (23.5)		8 (33.3)
*Other*		6 (17.6)		3 (12.5)
Type of main trauma						
*Interpersonal violence*		19 (55.9)		17 (70.8)	*χ*^*2*^ (3^c^) = 4.09	.252
*Witnessed harm to others*		3 (5.2)		0 (0)
*Accident*		11 (32.4)		5 (20.8)
*Other*		1 (2.9)		2 (8.3)
Months since trauma	39.43 (71.83)		49.67 (78.28)		t (56) = 2.59	.578
Number of previous traumas other than index event	2.38 (2.08)		2.96 (2.46)		t (56) = .57	.347
PDS pre-treatment	32.12 (8.29)		39.25 (8.25)		t (56) = 3.24	<.001^b^
PDS post-treatment	4.06 (4.44)		34.78 (10.13)		t (56) = 15.72^a^	<.001
Mean number of treatment sessions	12.15 (3.28)		13.58 (4.12)		t (56) = 1.49	.145
First session analysed		33 (97.1)		20 (83.3)	*χ*^*2*^ (1^c^) = 3.37	.067
Length of session analysed (mins)	85.65 (15.84)		77.46 (17.27)		t (56) = 8.19	.067^b^
Taking psychotropic medication		10 (29.4)		17 (70.8)	*χ*^*2*^ (1^c^) = 9.70	.002^b^
Comorbid Axis 1 Disorder		22 (64.7)		21 (87.5)	*χ*^*2*^ (1^c^) = 3.81	.051^b^
Comorbid Axis 2 Disorder		6 (17.6)		5 (20.8)	*χ*^*2*^ (1^c^) = 3.81	.760

*p < .05; **p < .01; ***p < .001.

a Equal variances not assumed.

b Significant group differences controlled for in ANCOVA.

c N = 58.

**Table 2:** Differences in early session between patients with good and poor treatment response.

Item	Good responders (*n* = 34)	Poor responders (*n* = 24)	ANCOVA^a^	*p*	Partial Eta Squared
*M (SD)*	*M (SD)*	*F (df)*
**In-session patient behaviours**					
Patient perseveration	3.44 (1.71)	4.13 (1.57)	F (1,51) = 5.26	.026	.093
Expression of thoughts and feelings	3.26 (1.19)	2.71 (1.16)	F (1,51) = 4.48	.039	.081
**Therapeutic relationship**					
Therapeutic alliance – agreement	49.29 (8.45)	45.29 (9.21)	F (1,51) = 4.05	.050	.073
Therapeutic alliance – relationship	17.82 (2.04)	17.75 (2.44)	F (1,51) = 0.88	.353	.017

a Controlling for initial PDS, comorbidity, medication, length of session, and marital status.

**Table 3:** Correlations between patient behaviours, therapeutic alliance, and treatment delivery.

	Patient behaviours		Therapeutic alliance		Treatment delivery	
Perseveration	Expression of thoughts and feelings	Agreement	Relationship	Competency	Efficiency
**Patient behaviours**						
Perseveration		.19	−.36**	.16	−.52***	−.49***
Expression of thoughts and feelings			.50***	.38**	.35**	.25
**Therapeutic alliance**						
Agreement				.69***	.55***	.24
Relationship					.49***	.16
**Treatment delivery**						
Competency						.66***
Efficiency						

*p < .05; **p < .01; ***p < .001.

## Footnotes

1 All patients meeting criteria for good response, and none of the poor response group, also met criteria for clinically significant change (Ehlers et al., 2013).

2 There were no differences in any demographic, diagnostic, trauma or treatment characteristics between eligible patients for whom working tapes were identified and those for whom tapes were no longer working/missing.

3 Available from corresponding author.
